# Advancing Treatment in Atopic Dermatitis: A Comprehensive Review of Clinical Efficacy, Safety, and Comparative Insights Into Corticosteroids, Calcineurin Inhibitors, and Phosphodiesterase-4 Inhibitors as Topical Therapies

**DOI:** 10.7759/cureus.55393

**Published:** 2024-03-02

**Authors:** Tyler D Hernandez, Sarah J Aleman, Maria Bao-Loc-Trung, Michael V Forte, William Brandt, Catherine Armstrong, Jeffrey Howard, Chizoba N Mosieri, Shahab Ahmadzadeh, Giustino Varrassi, Sahar Shekoohi, Alan D Kaye

**Affiliations:** 1 School of Medicine, Louisiana State University Health Sciences Center New Orleans, New Orleans, USA; 2 School of Medicine, Louisiana State University Health Sciences Center Shreveport, Shreveport, USA; 3 Department of Anesthesiology, Louisiana State University Health Sciences Center Shreveport, Shreveport, USA; 4 Pain Medicine, Paolo Procacci Foundation, Rome, ITA

**Keywords:** phosphodiesterase-4 (pde4) inhibitor, calcineurin inhibitors, topical corticosteroids, crisaborole, eczema, atopic dermatitis

## Abstract

Atopic dermatitis (AD) is a pervasive and multifaceted dermatological disorder causing daily distress to afflicted individuals worldwide. This comprehensive review synthesizes the historical and contemporary advancements in therapeutic strategies, offering a critical analysis of their efficacy, safety profiles, and adaptability. The enduring role of topical corticosteroids in managing AD is examined, acknowledging their potent anti-inflammatory properties alongside their potential adverse side effects, particularly in extended usage. The article explores the utilization of topical calcineurin inhibitors like tacrolimus and pimecrolimus, highlighting their novel anti-inflammatory pathways while also scrutinizing concerns over potential malignancies that relegate them to second-line therapy. The present investigation features the emergence of crisaborole, a phosphodiesterase four inhibitor. Its innovative mode of action, benign safety profile, and applicability to mild and moderate AD are thoroughly evaluated. The review also includes challenges, particularly cost considerations, which constrain accessibility and necessitate nuanced implementation in therapeutic regimens. This study underscores the need for persistent investigation, teamwork, and innovations in managing AD. In this regard, AD requires a united approach between clinicians, researchers, affected individuals, and policymakers to refine patient-focused treatment and develop precise, economical strategies to address this chronic and frequently life-altering health condition.

## Introduction and background

Atopic dermatitis (AD), a chronic, relapsing inflammatory skin disorder, plagues a substantial proportion of the global population, impacting the quality of life for both patients and caregivers. The clinical manifestations of this condition are pruritus and discomfort, ranging from mild, focal eczema to severe, widespread inflammation. Characterized by impaired skin barrier function, heightened immunological response, and susceptibility to skin infections, AD has become a topic of intense investigation and therapeutic development. The underlying pathogenesis involves intricate crosstalk among immune cells, keratinocytes, and various inflammatory mediators. Historically, AD management has primarily centered around topical corticosteroids, the cornerstone of therapy for moderate to severe manifestations [1]. While they effectively control acute symptomatic flares, the extended application of these compounds carries adverse risks locally, such as skin atrophy, cutaneous infection exacerbation, and comedone formation [2,3]. Topical calcineurin inhibitors (TCIs), including tacrolimus and pimecrolimus, are alternative therapies that have proven effective.

Despite recent evidence refuting these claims, concerns regarding a potential association with increased malignancy risk upon their advent have limited them to second-line treatment [4-8]. Given these issues, the quest for innovative, focused, and safer remedies led to the introduction of crisaborole, a phosphodiesterase-4 (PDE4) suppressant. Standing apart from corticosteroids and TCIs, crisaborole employs a distinctive mechanism, adjusting cyclic adenosine monophosphate (cAMP) concentrations in dermal cells, subsequently reducing pro-inflammatory markers like tumor necrosis factor-alpha (TNF-α) and T-cell mediators, such as interleukin-2 (IL-2) [9,10]. By targeting specific enzymatic pathways, crisaborole as a 2% topical ointment offers a refreshing alternative in the therapeutic armamentarium against mild to moderate AD [10,11].

The present investigation aims to provide an analysis of the physiological and pharmacological basis of treating AD with crisaborole compared to traditional topical steroids and calcineurin inhibitors. Exploration of the mechanism of action, route of administration, dosing, safety profile, and effectiveness of these therapies is provided. The review also addresses cost considerations, which pose a significant barrier to wide adoption despite its promising attributes [12,13]. Furthermore, a comparison of these therapies is presented by examining clinical studies, systematic reviews, and meta-analyses, offering insight into the evolving landscape of AD management. Emphasis is placed on the position of crisaborole in sequential therapy for mild to moderate AD and its suitability for application on delicate skin regions, such as the face, intertriginous areas, and genitals, where other therapies may be restricted [14,15].

By juxtaposing crisaborole with existing treatments, this review strives to delineate its potential as a transformative therapeutic approach, fostering a nuanced understanding of its role in the ever-changing paradigm of AD care. Furthermore, it aims to stimulate deeper clinical investigation into refining management strategies of AD, enhancing patient-focused care in this intricate and multifaceted dermatological condition.

## Review

Treating AD with crisaborole (PDE4 inhibitor)

Crisaborole is a PDE4 inhibitor and a treatment for AD [12]. The PDE4 enzyme plays a crucial role in the hydrolysis of cyclic adenosine monophosphate (cAMP) to AMP. By inhibiting PDE4, crisaborole increases cAMP levels in skin cells, which modulates inflammatory and immune responses [9]. Elevated cAMP causes downregulation of pro-inflammatory and T cell cytokines, such as tumor necrosis factor-alpha (TNF-α) and interleukin-2 (IL-2), respectively, thereby reducing inflammation in the affected skin [10]. This unique mode of action makes crisaborole an attractive therapeutic option for managing mild to moderate AD. Crisaborole is formulated as a 2% ointment for topical application and is used for the treatment of mild to moderate AD [10]. It is applied twice daily to the affected areas of the skin. The ointment is suitable for patients aged two years and older [10]. This easy-to-use topical formulation offers patients convenience and allows targeted application to affected skin areas.

Clinical studies indicate that crisaborole is generally well-tolerated with a favorable safety profile. Most observed adverse effects were mild to moderate and transient, mainly affecting the application site. These local reactions included pain, burning, and pruritus but did not typically necessitate treatment discontinuation [12]. Crisaborole has minimal systemic absorption, reducing the risk of systemic side effects commonly associated with corticosteroids and calcineurin inhibitors. As with any medication, caution is advised when using crisaborole in patients with known hypersensitivity to the drug or its components [13,14]. Crisaborole offers several advantages over traditional topical steroids and calcineurin inhibitors, making it a valuable addition to the drugs available for AD treatment. Crisaborole's safety profile distinguishes it from topical steroids and calcineurin inhibitors.

While corticosteroids effectively reduce inflammation, prolonged use is correlated with a wide range of side effects, including skin atrophy, telangiectasia, and hypothalamic-pituitary-adrenal axis suppression. Likewise, the application of topical steroids to facial or intertriginous areas can result in skin atrophy and is often contraindicated [11]. Calcineurin inhibitors, such as tacrolimus and pimecrolimus, are effective AD treatments, but the risk of skin cancer and lymphoma restricts their use [15]. Due to its minimal systemic absorption and favorable side effect profile, crisaborole is a safer option for long-term use, particularly for children and sensitive skin areas [11]. As there is no risk of dermal thinning or atrophy, crisaborole is suitable for delicate skin regions [15]. Treatment of mild to moderate AD with crisaborole represents a promising therapeutic approach, but further research will continue to define its optimal role in management. 

Treating AD with topical steroids

Topical corticosteroids are currently recommended as first-line therapy for acute control of moderate to very severe AD. The mechanism of action of topical steroids includes suppression of the quantity and activity of inflammatory cells and cytokines, such as neutrophils, lymphocytes, monocytes, Langerhans cells, interleukins, tumor necrosis factor, and granulocyte-monocyte colony-stimulating factor. They also induce the production of anti-inflammatory proteins, such as lipocortin, vasoregulin, and vasocortin. The vehicle options of topical corticosteroids include ointments, creams, lotions, gels, and foams [1]. Ointments are indicated for thick hyper-keratotic lesions and are the most potent, followed by creams, lotions, gels, and aerosols [16]. The vasoconstrictor assay is the gold standard for determining topical corticosteroid potency [1]. Topical corticosteroids are more permeable and better absorbed in regions of thin epidermis, including the eyelid, as opposed to thicker regions like the sole, with the penetration difference varying 300-fold [16].

Topical corticosteroids' most common local adverse effects include striae, atrophy, rosacea, perioral dermatitis, acne, and purpura [2]. A longer length of usage, a more potent vehicle, and a thinner site of application may increase the risk of these local and systemic adverse effects. Systemic adverse effects include suppression of the hypothalamic-pituitary-adrenal axis, which can affect the linear growth of children and bone density of adults, glaucoma, Cushing syndrome, hypertension, and hyperglycemia [2]. Topical steroids are contraindicated in patients with active bacterial or fungal infections, which include impetigo, furuncles, carbuncles, cellulitis, erysipelas, lymphangitis, and erythrasma. The anti-inflammatory and vasoconstrictive corticosteroid properties can mask symptoms of infection, thus delaying diagnosis and treatment [2].

A systematic review and meta-analysis of randomized clinical trials determined that calcineurin inhibitors and corticosteroids have similar efficacy. However, calcineurin inhibitors were associated with higher costs and more adverse effects, including skin burning and pruritis. The findings provide level-1a support for using corticosteroids as the therapy for AD, and calcineurin inhibitors should be used as second-line treatment [3]. However, topical corticosteroids are limited by cutaneous and systemic side effects when used inappropriately. A meta-analysis determined that nonsteroidal PDE4 inhibitors, such as crisaborole, are safer than topical corticosteroids. The most common adverse effects of crisaborole reported were mild application-site burning, pain, and stinging. Serious adverse effects were rare and considered not to be associated with treatment. This study concluded that crisaborole has the potential to be a safe and effective maintenance treatment option for mild to moderate AD. [17]. Another study found that crisaborole ointment was well-tolerated when applied to thin and sensitive skin areas, such as the face, intertriginous areas, and genitals. Meanwhile, the use of topical corticosteroids on thin-skinned regions is limited by adverse effects. Therefore, crisaborole may be more desirable in patients with AD in these areas [14]. A drawback of crisaborole is its price compared to topical corticosteroids. The current average wholesale price of crisaborole is approximately $10 per gram, while corticosteroids are several cents per gram. Although crisaborole is effective with a lower incidence of adverse events, this study concluded that crisaborole is significantly more expensive and should remain as second-line therapy after topical corticosteroids [12].

Treating AD with topical calcineurin inhibitors 

Topical tacrolimus and pimecrolimus are currently second-line calcineurin inhibitors approved for topical use in adults and children greater than two years of age with AD [4]. Topical tacrolimus is an anti-inflammatory immunomodulator that binds to a specific cytoplasmic immunophilin known as FKBP12. The subsequent tacrolimus/FKBP-12 complex further complexes with calcium, calmodulin, and calcineurin, preventing calcineurin's phosphatase action. This complex contains dephosphorylation and translocation of activated T-cells (NFAT) nuclear factor to the nucleus, effectively preventing gene transcription of interleukin-2 (IL-2) and other lymphokines, such as gamma interferon-downregulation of IL-2 signaling results in diminished T-cell activation, inflammatory cytokine release, and mononuclear cell infiltration. Tacrolimus may also hinder the accumulation of several other cytokines, such as IL-3, IL-4, tumor necrosis factor-alpha (TNF-α), and granulocyte-macrophage colony-stimulating factor (GM-CSF). In addition to the aforementioned anti-inflammatory effects, there is also an indication that tacrolimus downregulates FcεRI on Langerhans cells and hinders the release of pre-formed inflammatory cytokines from mast cells and basophils nested in the dermis [5]. Pimecrolimus's mechanism of action involves its ability to selectively inhibit the activation and subsequent inflammatory cytokine production of T-lymphocytes and mast cells. Pimecrolimus works through a similar mechanism as tacrolimus by binding to FKBP12, blocking both Th1 (IL-2, interferon-gamma) and Th2 (IL-4, IL-10) cytokines [18,19]. Topical tacrolimus is applied to the skin as a 0.03-0.1% ointment only in the affected areas of AD with careful avoidance of the eyes. For adults, adolescents, and pediatric patients, 0.03% or 0.1% ointment is applied to affected areas twice daily, approximately 12 hours apart [6,7,20]. Likewise, pimecrolimus is used in the affected area in adults or children with mild AD as a topical 0.1% cream twice daily for the affected areas of the skin [8].

Topical tacrolimus's most common adverse effects are a burning sensation, erythema, pruritis, and irritation at the application site. However, these effects are usually short-lived and diminish or resolve within a few days of initiating therapy. Unlike corticosteroids, tacrolimus does not cause skin atrophy or systemic effects [21]. Tacrolimus has not been proven to increase the risk of cutaneous infections, such as bacterial and viral infections, that often occur with AD. However, cutaneous infections typically seen more frequently in AD, such as *Staphylococcus aureus*, β-hemolytic *Streptococci*, herpes simplex, molluscum contagiosum, varicella, and eczema herpeticum, may still occur while on tacrolimus therapy [22-25]. For example, a phase IIIb study on the long-term safety of 0.03% and 0.1% tacrolimus ointment in children with AD aged two to 15 years proposed that the incidence rate of infections was similar to incidence rates in other pediatric studies assessing infection rates in AD patients and the general population [26]. In 2006, the United States Food and Drug Administration stated the unknown safety of prolonged calcineurin inhibitors along with a “black box” warning describing a potential risk of developing lymphoma and skin melanoma that has relegated these drugs to second-line therapy [27]. However, a systematic review and meta-analysis completed in 2023 found that the absolute risk of cancer with topical calcineurin inhibitor exposure was not increased compared to controls, and the rates of cancer comparatively between pimecrolimus and tacrolimus were similar [28]. Several clinical trials have shown the long-term safety of topical tacrolimus in dermal use for up to four years [5]. In addition, tacrolimus has been shown to have minimal functional effects on healthy skin in individuals with AD as opposed to other commonly used treatments [21].

Discussion

AD remains a complex and multifaceted dermatological condition affecting a significant portion of the global populace. As such, it is a necessity that management strategies undergo continual refinement to ensure quality care. This review underlines the evolution in therapeutic approaches, encapsulating the enduring reliance on topical corticosteroids, the utilization of topical calcineurin inhibitors, and the promising innovation of crisaborole, a PDE4 inhibitor. As the current first-line therapy, topical corticosteroids have been paramount in the management of AD due to their potent anti-inflammatory effects. However, their therapeutic potential is marred by a propensity for adverse side effects - particularly with prolonged usage [2]. The emergence of topical calcineurin inhibitors like tacrolimus and pimecrolimus presented a novel pathway to target inflammation, but, despite recent data suggesting otherwise, the “black box” carcinogenic association has relegated them to second-line therapy [4-8]. Crisaborole stands out as a refreshing alternative, given its distinct mode of action, favorable safety profile, and adaptability for mild to moderate AD. Its capacity to modulate cAMP levels and reduce pro-inflammatory cytokines marks a shift in AD treatment, fostering precision and minimizing systemic risks [9,10]. In addition, the lack of adverse effects commonly associated with corticosteroids and calcineurin inhibitors validates its suitability for delicate skin regions and extended use [11,13]. This convenience and tolerability, combined with its minimal systemic absorption, render it a valuable addition to AD care [12,13]. However, the therapeutic landscape is not devoid of challenges. Cost considerations continue to impede the broad accessibility of crisaborole, with its substantially higher price limiting it to specific situations or secondary options following topical corticosteroids [12]. While offering tangible benefits, the economic implications may necessitate a nuanced evaluation of its role in therapy, aligning it with individual patient needs and clinical context (see Tables 1, 2).

**Table 1:** Clinical efficacy and safety of PDE4 inhibitors and topical corticosteroids AD: atopic dermatitis; CI: confidence interval; HPA: hypothalamic-pituitary-adrenal; ISGA: Investigator’s Static Global Assessment; PDE4: phosphodiesterase-4; RCT: randomized controlled trial; RR: risk ratio

Author (Year)	Study design and population	Intervention	Efficacy	Safety	Conclusions
Paller et al. (2016) [29]	Two double-blind RCTs including patients aged two to 79 with AD and an ISGA score of 2 (mild) to 3 (moderate)	Crisaborole 2% ointment vs. vehicle placebo twice daily for 28 days	49% to 65% achieving ISGA 0 (clear) or 1 (almost clear) skin	Increased burning or stinging at application site with crisaborole (4.4%) vs. vehicle (1.2%)	Crisaborole exhibited minimal adverse effects and a rapid-onset of lasting amelioration of AD severity
Martín-Santiago et al. (2022) [13]	Meta-analysis of 16 RCTs including patients aged two to 79 with mild to moderate AD	Topical PDE4 inhibitors vs. vehicle placebo	Reduced rate of atopic dermatitis exacerbation (RR = 0.62; 95% CI 0.39-0.98; p = 0.04)	Pain at the application site (RR = 2.59; 95% CI 1.27–5.28; p = 0.01)	Topical PDE4 inhibitors are generally safe and well-tolerated in treating atopic dermatitis.
Zane et al. (2016) [14]	Double-blind RCT including 32 healthy volunteers aged 18 to 55	Crisaborole 2% ointment applied to 13 sensitive skin areas twice daily for 21 days	Well-tolerated in 98.8% of assessments	One subject reported application site pain that resolved in less than one day	Crisaborole ointment is suitable for delicate skin regions.
Coondoo et al. (2014) [2]	Literature review of numerous populations affected by psoriasis, eczema, and acne	Topical steroids of varying potency and usage patterns (e.g., frequency and duration)	Topical steroids have improved the therapy of inflammatory cutaneous disorders.	The rise in misuse is causing a higher frequency of local (skin atrophy and infection) and systemic (HPA axis suppression and psychological) adverse effects.	Long-term use of topical steroids should be approached with caution due to the myriad of potential side effects.

**Table 2:** Comparative studies of PDE4 inhibitors, calcineurin inhibitors, and topical corticosteroids in the pharmacological management of atopic dermatitis AD: atopic dermatitis; PDE4: phosphodiesterase-4; RCT: randomized controlled trial

Study design and population	Intervention	Results and findings	Conclusions
Thom et al. (2022) [15]	Matching-adjusted indirect comparison including patients aged >2 years with mild-to-moderate AD method	Comparison of crisaborole 2% with pimecrolimus 1%, tacrolimus 0.03%, and tacrolimus 0.1% over six weeks	Crisaborole 2% is more effective than pimecrolimus 1% or tacrolimus 0.03% in patients above two years of age with mild-to-moderate AD.
Broeders et al. (2016) [3]	Meta-analysis of 13 RCTs including children aged >2 to adults with moderate to severe atopic dermatitis	Comparison of topical calcineurin inhibitors with topical corticosteroids	Calcineurin inhibitors and corticosteroids showed similar efficacy in reducing the symptomology of AD, but calcineurin inhibitors were associated with higher costs and more adverse events, such as skin burning and pruritus.
Yang et al. (2019) [17]	Meta-analysis of seven RCTs, including patients aged two to 75 with mild to moderate AD	Topical PDE4 inhibitors vs. vehicle placebo over a treatment duration of 14 to 28 days	PDE4 inhibitors showed a statistically significant improvement in attaining clear or almost clear skin compared to vehicle. In addition, PDE4 inhibitors demonstrated fairly negligible adverse effects and can be considered safer than topical corticosteroids and topical calcineurin inhibitors in the treatment of AD.
Paller et al. (2005) [8]	Three investigator-blinded RCTs including children aged >2 to adults with mild to very severe AD	Comparison of tacrolimus ointment and pimecrolimus cream with application twice daily for six weeks	The tacrolimus ointment demonstrates superior efficacy and quicker therapeutic action and maintains a similar safety profile when compared to pimecrolimus cream for treating both adult and pediatric patients with AD.

## Conclusions

The synergy of conventional treatments and new approaches, such as crisaborole, provides a more enriched comprehension of AD therapies. This dynamic model highlights the importance of continuous research, clinical studies, and innovation in AD management. The cooperation of clinicians, researchers, patients, and policymakers will help to translate these developing scientific insights into practical and patient-centered care strategies. Such synergistic efforts are paramount in achieving optimal treatment results and raising the quality of dermatological care for AD. Further investigation of crisaborole may lead to more targeted and cost-effective medications for this prevalent and chronic disease. Further studies are expected to continue to explore the full therapeutic capacity of crisaborole, aiming to diversify the treatment options available. These advancements are critical to ameliorating the damage caused by AD, ensuring a brighter future for those suffering from this disease. The journey toward attaining these goals emphasizes our collective responsibility to foster innovation and to weave novel evidence into clinical decision-making as advocates for the well-being of our patients.
